# Enhancement of astaxanthin accumulation using black light in *Coelastrum* and *Monoraphidium* isolated from Malaysia

**DOI:** 10.1038/s41598-021-91128-z

**Published:** 2021-06-03

**Authors:** Marshila Kaha, Koji Iwamoto, Nurul Ashyikin Yahya, Noraiza Suhaimi, Norio Sugiura, Hirofumi Hara, Nor’Azizi Othman, Zuriati Zakaria, Kengo Suzuki

**Affiliations:** 1 Department of Chemical and Environmental Engineering, Malaysia-Japan International Institute of Technology, Universiti Teknologi Malaysia, Jalan Sultan Yahya Petra, 54100 Kuala Lumpur, Malaysia; 2 Department of Mechanical Precision Engineering, Malaysia-Japan International Institute of Technology, Universiti Teknologi Malaysia, Jalan Sultan Yahya Petra, 54100 Kuala Lumpur, Malaysia; 3 Euglena Co., Ltd, Tokyo, 108-0014 Japan; 4 Microalgae Production Control Technology Laboratory, RIKEN, Yokohama, Kanagawa 230-0045 Japan

**Keywords:** Natural variation in plants, Plant stress responses, Secondary metabolism

## Abstract

Microalgae are important microorganisms which produce potentially valuable compounds. Astaxanthin, a group of xanthophyll carotenoids, is one of the most powerful antioxidants mainly found in microalgae, yeasts, and crustaceans. Environmental stresses such as intense light, drought, high salinity, nutrient depletion, and high temperature can induce the accumulation of astaxanthin. Thus, this research aims to investigate the effect of black light, also known as long-wave ultraviolet radiation or UV-A, as a stressor on the accumulation of astaxanthin as well as to screen the antioxidant property in two tropical green algal strains isolated from Malaysia, *Coelastrum* sp. and *Monoraphidium* sp. SP03. *Monoraphidium* sp. SP03 showed a higher growth rate (0.66 day^−1^) compared to that of *Coelastrum* sp. (0.22 day^−1^). *Coelastrum* sp. showed significantly higher accumulation of astaxanthin in black light (0.999 g mL culture^−1^) compared to that in control condition (0.185 g mL^−1^). Similarly, *Monoraphidium* sp. SP03 showed higher astaxanthin content in black light (0.476 g mL culture^−1^) compared to that in control condition (0.363 g mL culture^−1^). *Coelastrum* sp. showed higher scavenging activity (30.19%) when cultured in black light condition, indicating a correlation between the antioxidant activity and accumulation of astaxanthin. In this study, black light was shown to possess great potential to enhance the production of astaxanthin in microalgae.

## Introduction

In recent years, there has been a surge in attention to extract bioactive compounds from natural resources due to their efficiency in the treatment of various diseases. Microalgae, which are oxygen generating photosynthetic microorganisms, are gaining great interest from biotechnological viewpoint. Microalgae can be classified as prokaryotic and eukaryotic organisms that are mostly found in the aquatic environment. These organisms possess unique characteristics of higher plants (oxygenic photosynthesis) combined with biotechnological attributes such as fast growth rates, ease of cultivation and the ability to accumulate primary and secondary metabolites^1,2^. These useful features led to the selection of microalgae in various applications such as biofuels, food, aquaculture, cosmetics, nutrition and pharmaceuticals.

Depending on the microalgae species, various valuable secondary metabolites can be extracted from its biomass, including carotenoids, polysaccharides, fatty acids and vitamins. Among all the secondary metabolites identified from microalgae, carotenoids have received particular attention due to their importance in therapeutic value, including antioxidant, neuroprotective, anti-angiogenic and anti-inflammatory activities^1,2^. Carotenoids comprise a huge group of natural compounds that are found predominantly in green plants, algae and some bacteria which belong to photosynthetic organisms^3^. It also can be found in non-photosynthetic microorganism such as fungi and yeast. Carotenoids are a class of terpenoid pigments consisting of carotenes (non-oxygenated molecule) and xanthophylls (oxygenated molecule) which can be distinguished based on their chemical structure^4^.

Among the carotenoids, astaxanthin, a group of xanthophyll carotenoids is recognized as one of the most powerful antioxidants found in nature that has been noted to surpass those of β-carotene and α-tocopherol (vitamin E)^5^. It is a red fat-soluble pigment found mainly in microalgae, yeast, and some crustaceans and gained a significant interest from pharmaceutical and nutraceutical industries due to the presence of two hydroxyl (OH) and two keto groups in the side chains of astaxanthin^6^. These applications include protection from ultraviolet (UV) light oxidation owing to astaxanthin’s anti-inflammatory and anti-aging properties^7^. Antioxidants are compounds that can neutralize the reactive oxygen species (ROS) and other free radicals that generated by various metabolic processes and environmental stresses such as smoke, air pollution and UV damage.

Production of astaxanthin can be carried out by two methods, namely biological synthesis and chemical synthesis^8^. Biological synthesis includes the production of natural astaxanthin from potential organisms such as microalgae, yeast and some crustaceans as a by-product, whereas chemical synthesis includes the production of synthetic astaxanthin from petrochemicals. Synthetic astaxanthin was not approved by the US Food and Drug Administration (FDA) for human consumption due to its low bioavailability and health issues^9^. Thus, production of natural astaxanthin is gaining interest as it is shown to have greater stability and more biological functions than synthetic astaxanthin.

In microalgae, astaxanthin is accumulated in the cytosolic lipid bodies (LBs)^10^ and acts as a defense chemical that reacts to environmental stresses such as nutrient starvation, high light intensity, high salinity, and high temperature. Light availability is one of the most important factors which control the photosynthesis process and production of carotenoids in the photosynthetic membrane vesicles^11^. In this study, black light was used to enhance the astaxanthin production in microalgae. Black light, also referred to as UV-A light, has a purple colour when it is turned on. UV light is classified into three wavelengths, UV-A (320–400 nm), UV-B (280–320 nm) and UV-C (200–280 nm)^12^. As the world is experiencing global warming and ozone-layer depletion^13^, UV radiation issue has become worse as it affects various organisms including humans and microalgae. UV radiation is harmful as it generates reactive oxygen species such as hydrogen peroxide (H_2_O_2_), superoxide anion (O_2−_) and hydroxyl radical (^∙^OH) which possess a strong oxidation ability^14^. However, UV radiation is considered to be a stressor for many physiological processes such as triggering the production of secondary carotenoids to protect the cells from damage^15^. Therefore, in this study, black light was used to increase the accumulation of astaxanthin in tropical microalgae isolated from Malaysia.

## Results

### Isolation of tropical microalgae and growth analysis

**Figure 1:**
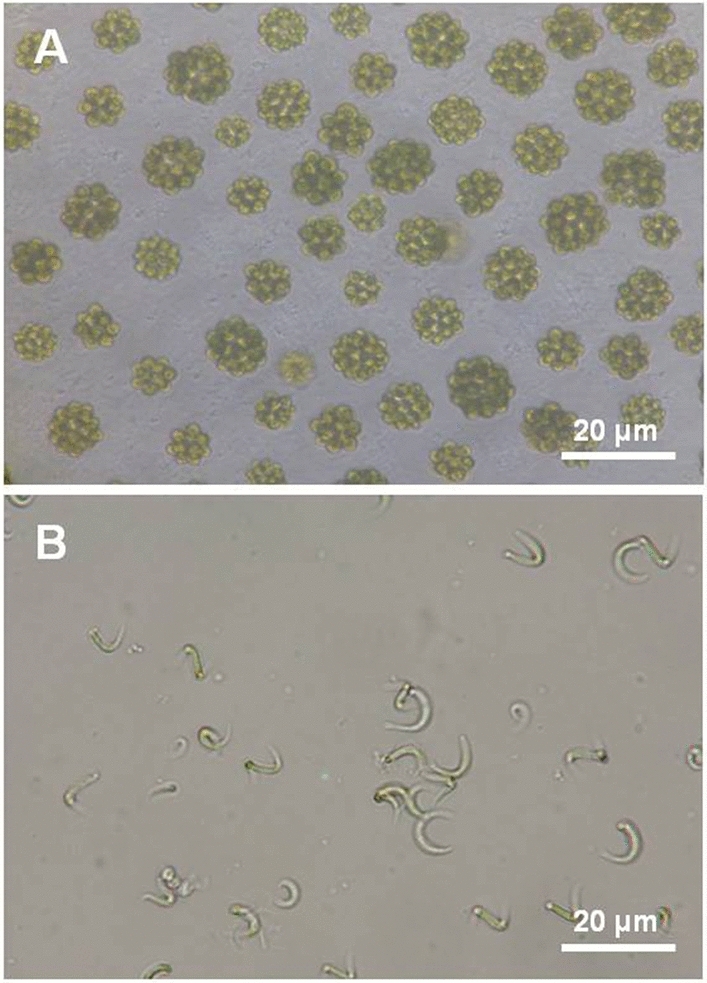
Light microscopic image with ×40 magnification. (**A**) *Coelastrum* sp., (**B**) *Monoraphidium* sp. SP3. Scale bar: 20 μm.

Algal strains used in this study were isolated from brackish water environment at Kuala Selangor Nature Park, Malaysia (3.361057, 101.244325). The pH of the collected water was 7.2 and the temperature at the sampling site was in a range of 27–29 °C. Prescreening was done by observing the microalgae under a microscope to monitor the growth and ability of the isolated species to accumulate carotenoids, which was indicated by the colour change (yellowish-orange, data not shown). From this screening, two out of ten isolated strains were chosen for further analysis. Their morphological characteristics are shown in Fig. 1. The alga in Fig. 1A was considered *Coelastrum* sp. because it showed typical morphology, ie. the spherical cells are arranged in pherical, about 30-celled coemobia^16^. The alga in Fig. 1B seemed to be *Monoraphidim* sp. from the morphology, a single cell with curved, sigmoid, or spiral, and gradually tapered towards the apex^17^. Strain SP03 was identified as a *Monoraphidium* representatives as it established a phyletic lineage associated with *Monoraphidium* strains in the 18S rRNA gene-based phylogenetic analysis (MW507790) (Fig. S1).

**Figure 2:**
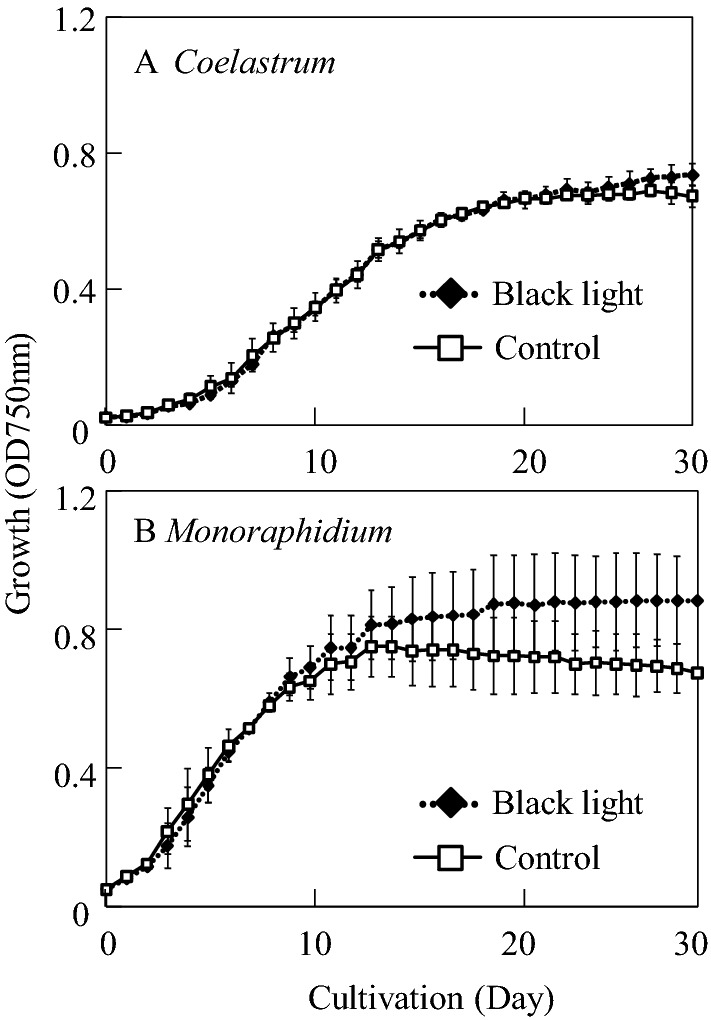
Algal growth in control and black light condition. (**A**) *Coelastrum* sp., (**B**) *Monoraphidum* sp. SP03; white square: control condition (continuous illumination of white fluorescene lamps);black square: black light condition (1 h illumination of black light with continuous illumination of white fluorescene lamps per day). Error bars correspond to the standard deviation of triplicate cultures.

Figure 2 shows the growth profiles of *Coelastrum* sp. and *Monoraphidium* sp. SP03 cultured in control (without black light) and black light conditions. In this study, the isolated strains were exposed to black light at different initiation times since the time to reach stationary phase of growth varied between different strains. *Coelastrum* sp. and *Monoraphidium* sp. SP03 were exposed to black light at day 13 and 9, respectively. *Monoraphidium* sp. SP03 culture of exhibited higher growth rate (0.66 day^−1^) compared to that of *Coelastrum* sp. culture (0.22 day^−1^) both under control and black light conditions as shown in Fig. 2B.

### Astaxanthin production in both strains cultured in black light condition

**Figure 3:**
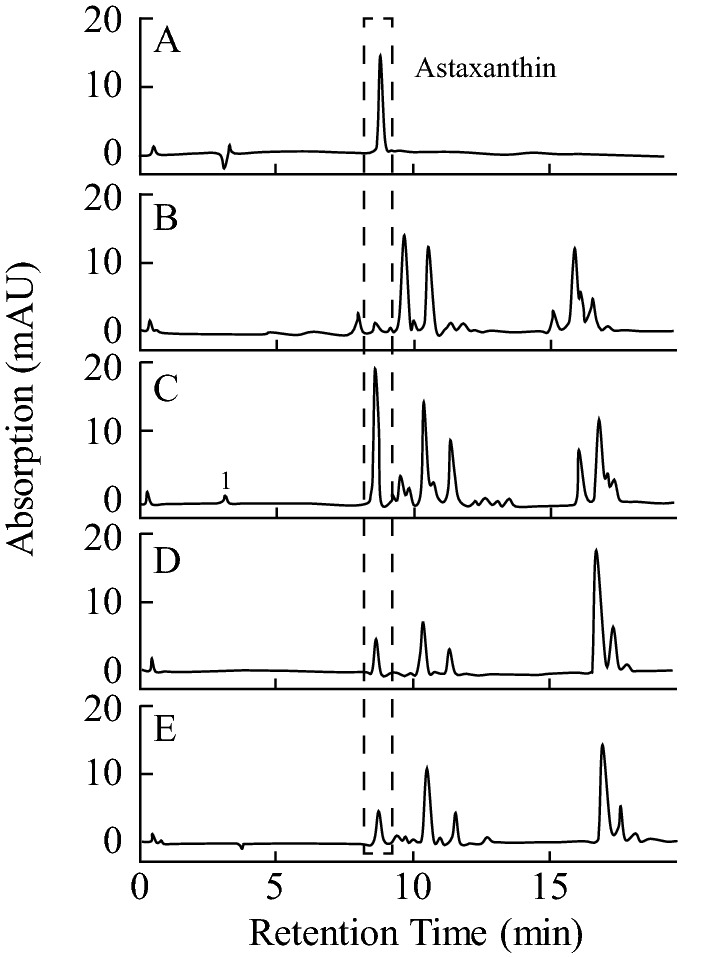
HPLC chromatogram of astaxanthin extracted after 30 days of cultivation. (**A**) astaxanthin standard, (**B**) *Coelastrum* sp. in control condition, (**C**) *Coelastrum* sp. in black light condition, **D** *Monoraphidium* sp. SP03 in control condition (**E**) *Monoraphidium* sp. SP03 in black light condition. Control condition: continuous illumination of white fluorescene lamps); Black light condition: 1 h illumination of black light with continuous illumination of white fluorescene lamps per day.

**Figure 4:**
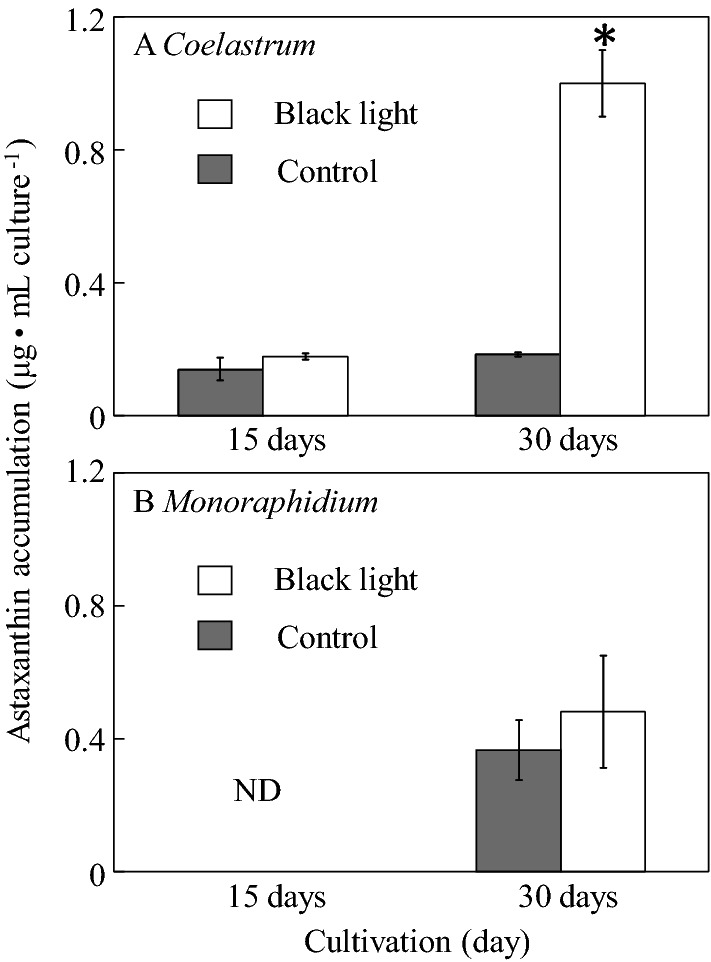
Astaxanthin production after 15 and 30 days of cultivation under black light and control conditions. (**A**) *Coealastrum* sp., (**B**) *Monoraphidum* sp. SP03. ND, not detected; white bar: black light condition; grey bar: control condition. Error bars correspond to the standard deviation of triplicate cultures. The asterisk shows that the p-value is less than 0.05.

Figure 3A shows the typical chromatogram and retention time for standard astaxanthin at 8.499 min (log *k’* = 1.35, Fig. S2), whereas Fig. 3B–E show the chromatograms of astaxanthin extracted from *Coelastrum* sp. and *Monoraphidium* sp. SP03 cultured in black light and control conditions after 30 days of cultivation. Both strains showed similar retention time and log *k’* to that of standard astaxanthin in either condition (Fig. 3, Fig. S2) with the absorbance maxima at 480 nm (personal communication from Dr. Tharek, A^18^, Fig. S3) and 474 nm^19^, respectively. The log *k’*s of contaminated carotenoids in *Coelastrum* sp. and *Monoraphidium* sp. SP03 in both control and black light conditions were shown in Fig. S2. Data of chromatogram obtained after 15 days of cultivation was not shown in this study. Figure 4 shows the production of astaxanthin in *Coelastrum* sp. and *﻿Monoraphidium* sp. SP03 after 15 and 30 days of cultivation. After 15 days, *Coelastrum* sp. cultured in black light and control conditions produced astaxanthin with the concentrations of 0.178 µg/mL culture and 0.139 µg/mL culture, respectively (Fig. 4A). Interestingly, when cultured in black light condition, the astaxanthin production in *Coelastrum* sp. increased remarkably (5.4-fold per volume culture) compared to that in control condition. The concentrations of astaxanthin in black light and control conditions were 0.999 µg/mL culture and 0.185 µg/mL culture, respectively. This difference was significant as verified statistically by using *t*-test (*p* < 0.05). In *Monoraphidium* sp. SP03, no production of astaxanthin was observed in either control or black light condition after 15 days of cultivation (Fig. 4B). After 30 days of culture, the production of astaxanthin was significantly increased (0.23-fold per volume culture) by black light compared to those cultivated in control condition. The concentrations of astaxanthin in black light and control conditions were 0.476 µg/mL culture and 0.363 µg/mL culture, respectively.

### Antioxidant profile of astaxanthin extracted from *Coelastrum* and *Monoraphidium*

**Figure 5:**
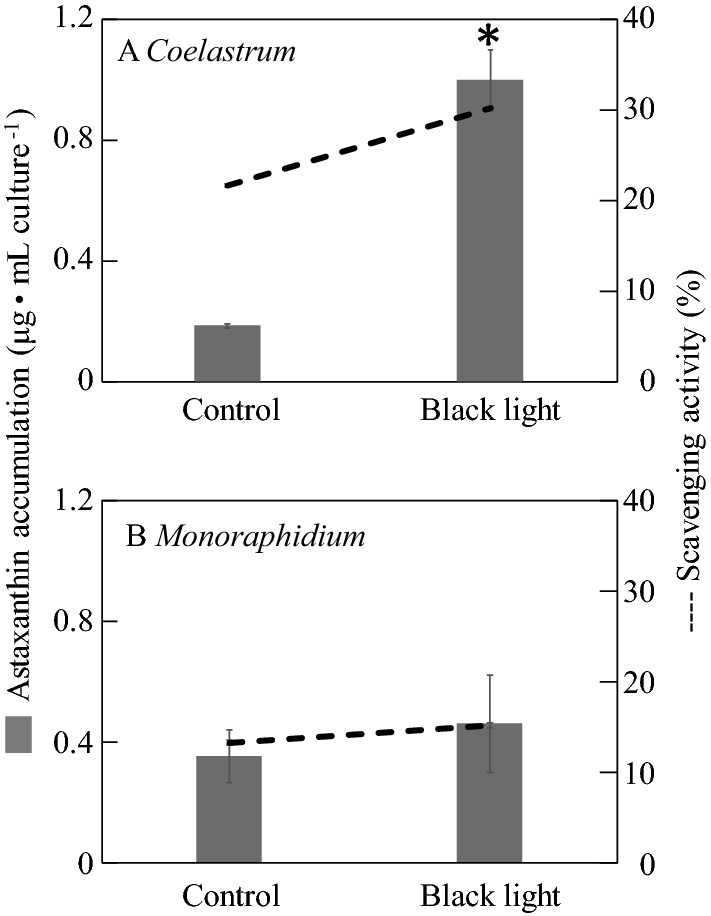
Astaxanthin production and scavenging activity in (**A**) *Coelastrum* sp. and (**B**) *Monoraphidum* sp. SP03 after 30 days of culture. Grey bar: astaxanthin content (µg mL culture^−1^); dashed line: scavenging activity. The asterisk shows that the p-value is less than 0.05.

Scavenging activity is the ability of an antioxidant to inhibit oxidation. Thus, the higher the scavenging activity, higher the ability of the antioxidant to scavenge the free radicals. Both *Coelastrum* sp. and *Monoraphidium* sp. SP03 cultured in black light condition showed higher radical scavenging activity compared to those cultured in control condition (Fig. 5). In this study, correlation analysis was conducted to test the relationship between the scavenging activity and the amount of astaxanthin produced. The results indicated that both *Coelastrum* sp. (Fig. 5A) and *Monoraphidium* sp. SP03 (Fig. 5B) showed a correlation between the production of astaxanthin and scavenging activity with r-values 0.75 and 0.99, respectively. The scavenging activity of astaxanthin extracted from *Coelastrum* cells was approximately 30.19% per volume culture, which was higher compared to that of *Monoraphidium* sp. SP03 This was consistent with the higher astaxanthin production by *Coelastrum* sp*.*

## Discussion

In the present study, *Coelastrum* sp. that belong to the family of *Scenedesmacea* and *Monoraphidium* sp. SP03 that comes from *Selenastraceae* family were successfully isolated from brackish water in Malaysia*.* The isolation of tropical strain would be the promising approach for the adaptation to local changing environment and can provide more productive culture. There were only few reports on the production of astaxanthin by these strains. A report by Liu et al.^20^ showed that *Coelastrum* sp. HA-1 produced 1.6-fold of astaxanthin as compared to control condition when induced with linoleic. Similarly, *Coelastrum* sp. TISTR 9501RE showed increase in carotenoid biosynthesis in nutrient-depleted condition^21^. The carotenoid production by *Coelastrum* cf. *pseudomicroporum* was also reported using urban wastewater^22^. More recently, astaxanthin production by *Coelastrum* sp. was shown to be enhanced by methyl viologen as the reactive oxygen species enhancer^18^. In case of *Monoraphidium* strain, previous study improved the photoautotrophic culture condition and outdoor cultivation method for increasing the astaxanthin content in *Monoraphidium* GK12^23,24^^.^ In the present study, black light was introduced to increase the accumulation of astaxanthin in *Coelastrum* sp. and *Monoraphidium* sp. SP03. These strains were subjected to a stress condition induced by the black light at their initial stationary phase of growth. Carotenogenesis has been reported to be enhanced when microalgae are subjected to unfavourable or stress conditions such as nutrient depletion, intense irradiation, high salinity or high temperature which usually occur when the algal cells reach the stationary phase^25^.

Astaxanthin was extracted from *Coelastrum* sp. and *Monoraphidium* sp. SP03 at day 15 and 30 and compared. Based on the results shown in Fig. 4, both strains showed higher astaxanthin production on day 30 compared to that on day 15. Both strains showed higher astaxanthin content in black light condition compared to those cultured in control condition. However, only *Coelastrum* sp. showed a significant difference in the amount of astaxanthin produced between the control and black light conditions, as verified by using *t*-test analysis (*p* < 0.05). On the other hand, *Monoraphidium* sp. SP03 cultured in control and black light conditions showed no statistically significant difference (*p* > 0.05).

Light intensity is one of the factors that influence the carotenogenesis in microalgae^26^. Photosynthesis can proceed at both low and high light intensities. Under low light intensity, fewer oxygen radicals are generated, whereas under high light intensity, surplus energy activates more oxygen molecules, since the cells are unable to utilize all energy that is generated by photosynthesis. The excess energy leads to the formation of astaxanthin^27^. Thus, the early culture stage of the strains resulted in low accumulation and no detection of astaxanthin due to exposure of the cultures to low light intensity (Fig. 3).

The black light, referred to as UV-A radiation, has a purple color when it is turned on. The term UV radiation is used to describe the UV wavelength region ranging from 200 to 400 nm and is classified into three categories, UVC (200–280 nm), UVB (280–320 nm) and UVA (320–400 nm)^12^. UV radiation exerts both positive and negative effects on microalgae cells^28^. For instance, UV radiation was known to cause DNA damage in the cells. Photodamage and conformational change caused by absorption of UV radiation by nucleic acids and proteins directly impair the vital metabolic functions such as DNA replication, translation, and transcription^29^. UV-A irradiation has been shown to inhibit photosynthesis^30^. Thus, it needs to be counteracted or avoided. Despite the inactivation of photosynthesis, the irradiation drives the accumulation of secondary metabolites^31^ and induced oxidative stress that resulted in the accumulation of carotenoids^32^.

At present, the effect of black light radiation on the accumulation of astaxanthin has not been reported. There were only a few reports on the effect of UV-A radiation on the production of carotenoids in microalgae. Salguero et al. studied the effect of adding UV-A radiation with different intensities to photosynthetically active radiation in marine microalga *Dunaliella bardawil*^33^. They observed that exposure to UV-A enhanced the production of total carotenoids, mainly β-carotene, lutein, and zeaxanthin, as well as the cell growth and the photosynthetic efficiency of *D. bardawil*. Higher β-carotene accumulation was observed in *D. bardawil* when UV-A radiation was coupled with nitrogen starvation^34^. The findings of the present study are consistent with the results of the previous studies which showed that UV-A could be one of the stressors that can enhance the accumulation of carotenoids in the algal cells.

Carotenoids show significant absorption in the UV-A spectrum, and this can be the logic behind the correlation between the higher production of astaxanthin (this study) as well as the higher production of β-carotene (previous studies) in microalgae and UV-A radiation (Figs. 3, 4). Astaxanthin has been known as an excellent antioxidant in numerous applications, for example, as an additive in feed industries, cosmetics as well as pharmaceuticals^35^. It has been reported that antioxidants play a role in preventing certain tumors as well as ameliorating degenerative and cardiovascular diseases^36^.

In this study, DPPH assay was performed as a fast screening method to analyze the antioxidant profile of astaxanthin extracted from *Coelastrum* sp. and *Monoraphidium* sp. SP03 after 30 days of culture. DPPH assay is a simple and easy method to perform antioxidant assays. In addition, DPPH is a stable radical compound that does not disintegrate in methanol, ethanol and water. Thus, in this study, ethanol was used as a solvent to dilute DPPH. DPPH assay has been successfully used to analyze the antioxidant properties of edible seed oils, wheat grains, and herbs^37^.

In this study, both *Coelastrum* sp. and *Monoraphidium* sp. SP03 showed a correlation between the production of astaxanthin and scavenging activity. *Coelastrum* sp. showed 30.19% scavenging activity that correlated with the higher amount of extracted astaxanthin. Generally, carotenoids in microalgae act as excellent antioxidants by antagonizing oxidative stress and nitrogen stress through neutralizing the excess of free radicals and ROS. In addition, strong antioxidants are commonly found in the carotenoids, especially from microalgae, due to their unique molecular structure, which consists of an additional oxygenic functional group (carbonyl, hydroxyl and epoxy) and several conjugated double bonds. Apart from astaxanthin, other carotenoids such as β-carotene, lutein, α-tocopherol, canthaxanthin, and fucoxanthin were also reported in microalgae due to their ability to scavenge free radicals. Nonetheless, the antioxidant properties of these carotenoids are not as strong as that of astaxanthin.

Astaxanthin is chemically similar to β-carotene but more powerful in terms of singlet oxygen quenching mechanism (antioxidant activity) compared to other members of carotenoids such as β-carotene, lutein, and lycopene^38^. The antioxidant activity of astaxanthin was tenfold higher than that of β-carotene and 500-fold higher than that of α-tocopherol^39^. A number of studies have validated the superior antioxidant activity of astaxanthin. In particular, the potent antioxidant properties of astaxanthin, zeaxanthin, lutein, β-carotene, and lycopene were studied in specific physio-chemical interactions with membranes^40^. Their result showed that astaxanthin could suppress lipid peroxidation and maintain the membrane integrity, whereas lutein and β-carotene could not prevent the distortion of the membrane structure and production of high levels of lipid hydroperoxides. To summarize, astaxanthin plays an important role in scavenging the ROS produced by exposure to black light. In addition, *Coelastrum* sp. showed astaxanthin accumulation following exposure to black light. To clarify the effect of black light on the production of axtaxanthin in algae, further study would be required such as the comparative analysis of astaxanthin production among *Coelastrum* sp., *Heamatococcus,* and other algal strains with black light.

## Materials and methods

### Isolation and culture conditions

Water samples were collected from a freshwater environment at Kuala Selangor Nature Park, Selangor Malaysia (3.361057, 101.244325) by using 25 µm plankton net from a known distance. The pH of water samples was measured by using a compact pH meter (B-71X, Horiba Scientific, Japan), while the temperature of water samples and surroundings were measured by Thermo recorder TR-71wf (T&D, Japan). Microalgae cells from the water samples were isolated using traditional isolation technique by picking up the single cells using a Pasteur pipette^41^. This technique included the use of a Pasteur pipette attached to a latex tube and the isolation process was performed under a microscope (Eclipse TS100, Nikon, Japan). A drop of sterilized AF-6 medium^42^ was placed on a glass slide and into the microplate. A single cell of microalgae from the collected water samples was transferred to the AF-6 medium on the glass slide. The isolated cells were washed several times (2–3 times) on the washing plate before being transferred to the 96-well microplates containing AF-6 medium. The microplates were incubated at (28 ± 1) °C. Throughout this process, sterilized equipments and media were used to avoid contamination and to get unialgal cultures.

Isolated culture (5 mL) was transferred to 250 mL Erlenmeyer flask containing 100 mL of medium (5% v/v inoculation). The Erlenmeyer flask was placed on an orbital shaker (Orbitron, InforsHT, Switzerland) with the speed of 120 rpm at (28 ± 1) °C under continuous illumination by white fluorescene lamps (18 W/865, TL-D, Philips, Nederland) at 35 ± 5 µmol photon m^−2^ s^−1^. After the cultures reached stationary phase, the light intensity was increased to 165 ± 5 µmol photon m^−2^ s^−1^ with continuous illumination. All inoculations were prepared in triplicate.

### Exposure to black light

To investigate the effect of black light on the production of astaxanthin, triplicate culture of each strain was prepared with 5% v/v inoculation. These triplicate cultures were maintained in identical conditions until they reached the early stationary phase. The cultures were subjected to continuous illumination (165 ± 5 µmol photon m^−2^ s^−1^) and black light (365 nm, 18 W, TL-D, Philips, Nederland). The triplicate cultures were exposed to black light for 1 h every day.

### Growth measurement

The optical density at 750 nm was measured every day by using a colorimeter (Spectronic 20A, Shimadzu, Japan). The specific growth rate was calculated using the Eq. (1)^43^

1 $${\text{Specific growth rate:}}\; \,\upmu = ({\text{lnOD}}_{{{\text{t}}2}} {-}{\text{lnOD}}_{{{\text{t}}1}} )/({\text{t}}_{2} - {\text{t}}_{1} )$$

where OD_t2_ = Highest absorbance (OD_750_) at exponential phase, OD_t1_ = Lowest absorbance (OD_750_) at exponential phase, t_2_ = time (day) at OD_t2_, t_1_ = time (day) at OD_t1_.

### Phylogenetic analysis

Plant DNA Preparation Kit (Jena Bioscience, Germany) was employed to extract the genomic DNA with slight modification of the manufacturer’s protocol. Two eukaryotic universal primers to amplihy the specific region of nuclear-encoded small subunit ribosomal RNA gene (18S rDNA), SR1 (forward, 5ʹ-TACCTGGTTGATCCTGCCAG-3ʹ) and SR12 (reverse, 5ʹ-CCTTCCGCAGGTTCACCTAC-3ʹ) were used^44^. PCR mastermixes were prepared by using Promega Kit (Promega, USA). The PCR reaction conditions were as described; 30 cycles of initial denaturation at 94 °C for 2 min, denaturation at 98 °C for 10 s, annealing at 53 °C for 30 s, and extension at 72 °C for 2 min. Amplified DNA was sequenced by First Base Laboratory Sdn. Bhd. (Kuala Lumpur, Malaysia), and the sequences were analysed by BLAST algorithm available on National Center for Biotechnology Information (NCBI) website. Genbank database sequecnces having 90–99% similarity With the analyzed sequence were aligned by using MEGA X software. The phylogenetic tree was constructed using Maximum likelihood method with bootstrap test (1000 replicates).

### Extraction of astaxanthin

Astaxanthin from the isolated microalgae was extracted by the acid-acetone method as described previously^45^. The samples (10 mL) were centrifuged, and the harvested biomass was lyophilized and used for the extraction process. The biomass was treated with 1 mL of 1 N HCl (Sigma-Aldrich, USA) at 70 °C for 45 min. The sample was cooled at room temperature (25 °C) and centrifuged (10,000*g*) for 10 min. Then, the treated sample was washed twice with deionized water (1 mL) and suspended in 1 mL of acetone (Sigma-Aldrich) prior to incubation for 1 h in an orbital shaker (200 rpm). The sample was centrifuged (10,000*g*) for 5 min. The supernatant was filtered through 0.45 µm membrane filters (Whatman, Germany) and was injected into vials for high-performance liquid chromatography (HPLC) analysis.

### Quantification of astaxanthin content

The astaxanthin content was analyzed by HPLC system (1220 Infinity LC, Agilent Technologies, USA) with a diode-array detector (DAD, threshold 0.001 mAU). The chromatographic separation was achieved on Eclipse Plus C18 column (5 µm, 4.6 × 250 mm, Agilent Technologies). The extracts (10 µL) were injected onto the column with 0.8 mL/min flow rate. The optical density at 475 nm detected by DAD with 4 nm of slit width and 4 nm band width was used for quantifying astaxanthin. The mobile phase consisted of solvent A (acetone HPLC grade) (Sigma-Aldrich) and solvent B (methanol: H_2_O, 9:1) (Sigma-Aldrich) with the following 30 min gradient: 0 min (80% solvent B, 20% solvent A), 10 min (50% solvent A, 50% solvent B), and 20 min (20% solvent B, 80% solvent A). Standard astaxanthin (Sigma-Aldrich) was used to compare the concentrations of astaxanthin in isolated algal samples. The peaks were identified from the retention time and log *k’*^46^.

### Antioxidant activity assay

The DPPH assay was performed as described previously^47^. The antioxidant activity was assessed colorimetrically at 517 nm. The reagent 1, 1ʹ diphenyl-2-picrylhydrazyl (DPPH) (Sigma-Aldrich) (2.4 mg) was mixed with ethanol (100 mL) and stored at 20°C in dark until further use. Then, sample (0.2 mL) was added to ethanol (2 mL), incubated for 20 min, and assessed as a diluted sample. For the blank, distilled water (0.2 mL) was added to ethanol (2 mL) and incubated for 20 min. Both dilutions were stored at –20°C in dark until further use. Then, the diluted sample (1.5 mL) was mixed with DPPH solution (0.06 mM, 1.5 mL). This reaction was incubated for 30 min in dark at room temperature. This step was repeated for the blank solution. Finally, the solution was analyzed spectrophotometrically at 517 nm. The percentage of radical scavenging activity was was calculated using the Eq. (2).

2 $${\text{Radical scavenging activity}}\;\left( \% \right) = {\text{Abs of blank}} - \frac{{{\text{Abs of sample}}}}{{{\text{Abs of blank}}}} \times 100.$$

### Statistical analysis

All the assays used in this research were conducted in triplicates and the results were expressed as mean values. A two-tailed paired t-test was performed to check whether the utilization of black light could affect the production of astaxanthin by the microalgae. The *p*-value of less than 0.05 was used to indicate statistically significant differences^48^. In addition, correlation analysis was also conducted to check the linear relationship between the antioxidant profile and astaxanthin production in isolated species. Pearson’s correlation coefficient (r-value) was used to measure the correlation between the two variables, where the correlation coefficient value of 0.7 to 1.0 indicated a strong positive linear relationship. Microsoft Excel 2010 equipped with the Analysis Toolpak was used as a statistical tool.

## Supplementary Information

Supplementary Information.

## Data Availability

The datasets generated during and/or analysed during the current study are available from the corresponding author on resasonable request.
