# Supplementary material for: Enhancement of astaxanthin accumulation using black light in Coelastrum and Monoraphidium isolated from Malaysia
Source: Sci Rep. 2021 Jun 3;11:11708. doi: 10.1038/s41598-021-91128-z (PMC8175563; doi:10.1038/s41598-021-91128-z)
Supplement: Supplementary file 1 — Supplementary Information. [file 41598_2021_91128_MOESM1_ESM.pdf]

Enhancement of Astaxanthin Accumulation Using Black Light in *Coelastrum* and *Monoraphidium* Isolated from Malaysia

Marshila Kaha[1, +], Koji Iwamoto[1, +, *], Nurul Ashyikin Yahya[1], Noraiza Suhaimi[1], Norio Sugiura[1], Hirofumi Hara[1], Nor'Azizi Othman[2], Zuriati Zakaria[1], and Kengo Suzuki[3, 4]

[1] Department of Chemical and Environmental Engineering, Malaysia-Japan International Institute of Technology, Universiti Teknologi Malaysia, Jalan Sultan Yahya Petra, 54100 Kuala Lumpur, Malaysia

[2] Department of Mechanical Precision Engineering, Malaysia-Japan International Institute of Technology, Universiti Teknologi Malaysia, Jalan Sultan Yahya Petra, 54100 Kuala Lumpur, Malaysia

[3] euglena Co., Ltd., Tokyo 108-0014 Japan

[4] Microalgae Production Control Technology Laboratory, RIKEN, Kanagawa, 230-0045, Japan

*Corresponding author

E-mail address: k.iwamoto@utm.my

Tel: +60- 3-2203 1238; Fax: +60-3 2203 1266

[+] these authors contributed equally to this work

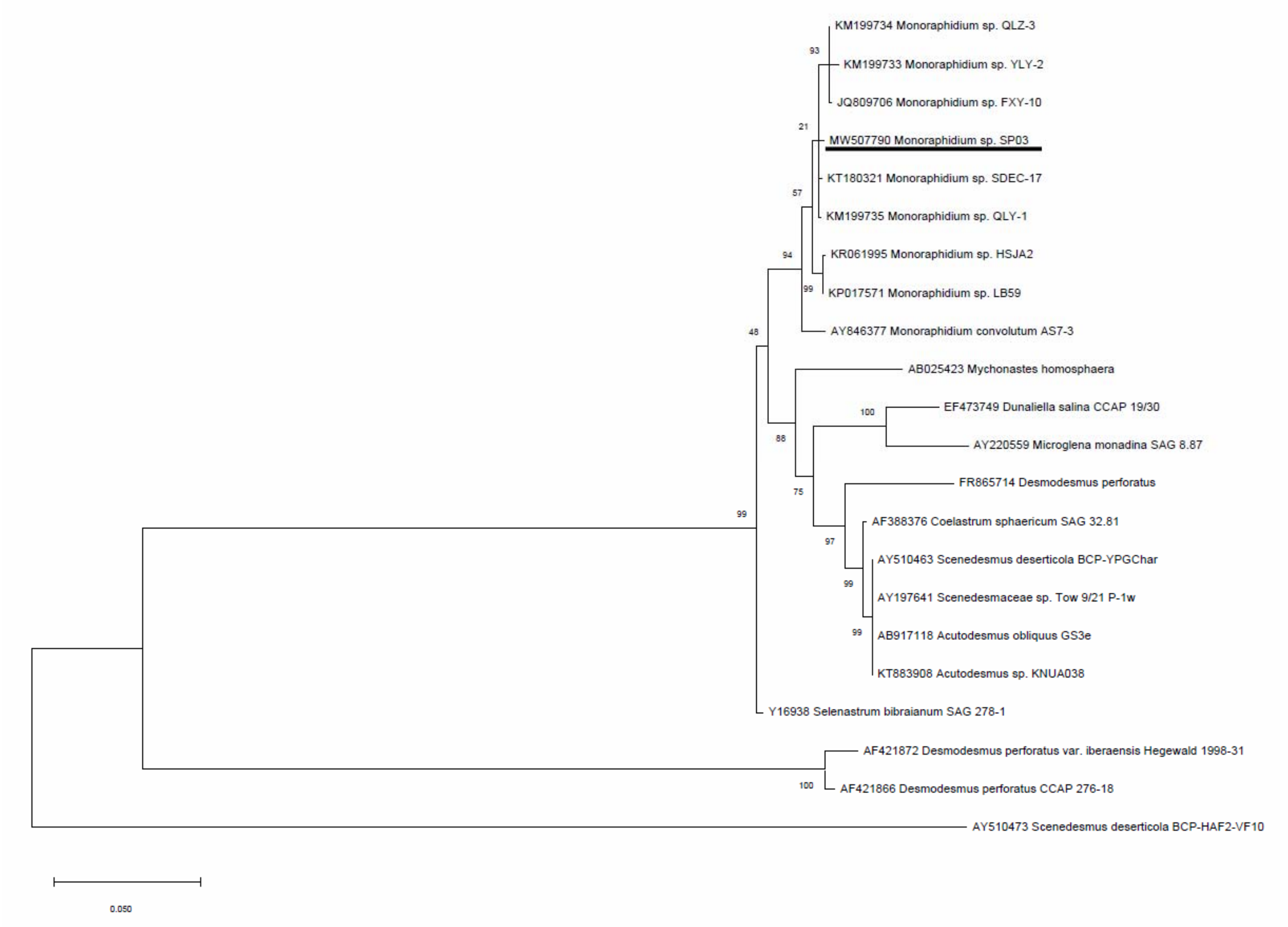

Fig. S1 Phylogenetic tree of *Monoraphidium* sp. SP03. Maximum likelihood method was used to construct the distance with the tree by MEGA X software.

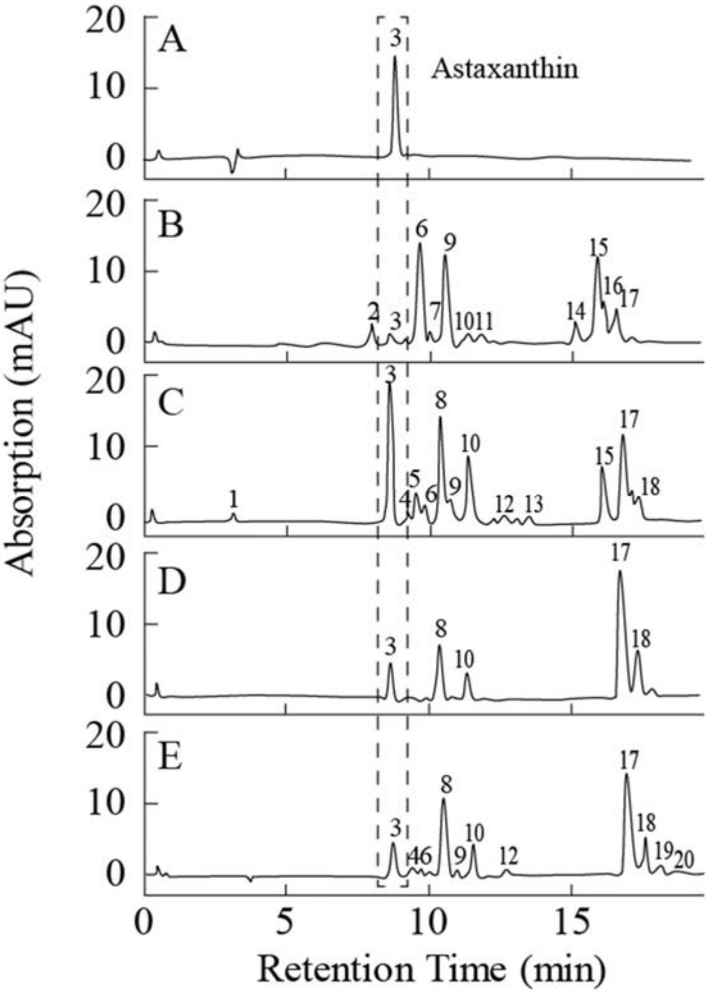

Peak	Log *k'*						
	A	B	C	D	E	pigment	absorbance maxima
1	-	-	0.91	-	-		
2	-	1.32	-	-	-		
3	1.35	1.35	1.35	1.35	1.35	Astaxanthin	*Coelastrum* 480 nm (personal communication from Dr. Tharek, A [18]), *Monoraphidium* 474 mm [19].
4	-	-	1.38	-	1.38		
5	-	-	1.39	-	-		
6	-	1.40	1.41	-	1.41		
7	-	1.42	-	-	-		
8	-	-	1.43	1.43	1.43		
9	-	1.44	1.44	-	1.44		
10	-	1.48	1.47	1.46	1.47		
11	-	1.49	-	-	-		
12	-	-	1.51	-	1.51		
13	-	-	1.54	-	-		
14	-	1.60	-	-	-		
15	-	1.62	1.62	-	-		
16	-	1.63	-	-	-		
17	-	1.64	1.64	1.63	1.63		
18	-	-	1.64	1.65	1.65		
19	-	-	-	-	1.66		
20	-	-	-	-	1.68		

Fig. S2. HPLC chromatograms of carotenoid A: Astaxanthin standard; B: *Coelastrum* in control condition; C: *Coelastrum* in black light condition; D: *Monoraphidium* sp. in control condition; E: *Monoraphidium* sp. in black light condition; -: not detected

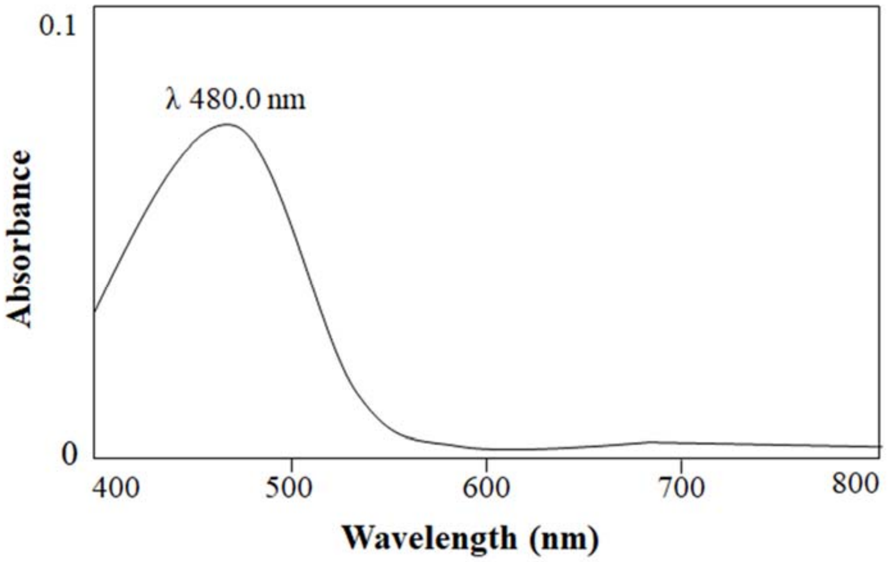

Fig. S3 Absorbance spectra of extracted astaxanthin from *Coelastrum* sp. (personal communication from Dr. Tharek, A [18])
